# AP2M1 Supports TGF-β Signals to Promote Collagen Expression by Inhibiting Caveolin Expression

**DOI:** 10.3390/ijms22041639

**Published:** 2021-02-06

**Authors:** Saerom Lee, Ga-Eun Lim, Yong-Nyun Kim, Hyeon-Sook Koo, Jaegal Shim

**Affiliations:** 1 Research Institute, National Cancer Center, 323 Ilsan-ro, Goyang-si 10408, Gyeonggi-do, Korea; 74419@ncc.re.kr (S.L.); 74621@ncc.re.kr (G.-E.L.); ynk@ncc.re.kr (Y.-N.K.); 2 Department of Biochemistry, Yonsei University, 50, Yonsei-ro, Seodaemun-gu, Seoul 03722, Korea

**Keywords:** AP2M1, *dpy-23*, endocytosis, TGF-β, caveolin, cuticle, *C. elegans*

## Abstract

The extracellular matrix (ECM) is important for normal development and disease states, including inflammation and fibrosis. To understand the complex regulation of ECM, we performed a suppressor screening using *Caenorhabditis elegans* expressing the mutant ROL-6 collagen protein. One cuticle mutant has a mutation in *dpy-23* that encodes the μ2 adaptin (AP2M1) of clathrin-associated protein complex II (AP-2). The subsequent suppressor screening for *dpy-23* revealed the *lon-2* mutation. LON-2 functions to regulate body size through negative regulation of the tumor growth factor-beta (TGF-β) signaling pathway responsible for ECM production. RNA-seq analysis showed a dominant change in the expression of collagen genes and cuticle components. We noted an increase in the *cav-1* gene encoding caveolin-1, which functions in clathrin-independent endocytosis. By knockdown of *cav-1*, the reduced TGF-β signal was significantly restored in the *dpy-23* mutant. In conclusion, the *dpy-23* mutation upregulated *cav-1* expression in the hypodermis, and increased CAV-1 resulted in a decrease of TβRI. Finally, the reduction of collagen expression including *rol-6* by the reduced TGF-β signal influenced the cuticle formation of the *dpy-23* mutant. These findings could help us to understand the complex process of ECM regulation in organism development and disease conditions.

## 1. Introduction

The extracellular matrix (ECM) provides essential structural scaffolding for cellular components and plays a critical role in various cellular responses such as cell proliferation, adhesion, migration, differentiation, and survival [1]. ECM is composed of extracellular macromolecules, including collagens, elastin, laminins, and fibronectin [2]. Among the components of ECM, collagen is the most abundant protein in the human body, accounting for about 30% of total human protein mass [3]. Collagen creates structural integrity and performs a variety of functions within tissues. Similarly, the simple model animal, *Caenorhabditis elegans*, has a complex ECM structure called a cuticle that covers the entire body. As an exoskeleton, the ECM plays an important function in the development of larvae, such as molting, and protection from the outside, such as pathogens and the environment [4,5]. As collagen is one of important components in the cuticle, *C. elegans* cuticle mutants exhibit a broad range of phenotypes such as the lethal (Let), abnormal embryogenesis (Emb), dumpy (Dpy), long (Lon) and roller (Rol) phenotypes [5].

Transforming growth factor-β1 (TGF-β1) is one of the most important regulators of collagen type I gene expression. TGF-β plays an important role in the development, homeostasis, and repair of all tissues in various organisms, from nematodes to humans [6]. For example, activation of TGF-β1 signaling induces the synthesis of ECM proteins and inhibits the degradation of matrix proteins, thereby contributing to the restoration of normal tissue structure for tissue repair [7]. However, chronic expression of TGF-β1 increases collagen accumulation, leading to excessive fibrosis [8]. Therefore, tight regulation of TGF-β signaling is critical for the tissue homeostasis of organisms.

The TGF-β signaling pathway is highly conserved at the molecular and functional levels in most animals. TGF-β receptors are transmembrane serine/threonine kinase and there are two types of TGF-β receptors. Upon TGF-β binding to the TGF-β type II receptor (TβRII), TβRII forms a complex with type I receptor (TβRI) and phosphorylates TβRI. Activated TβRI sequentially phosphorylates the receptor-regulated Smad (R-Smad), which then forms a complex with the common-mediator Smad (Co-Smad) and moves to the nucleus to regulate gene expression [6,9]. In *C. elegans,* collagen and TGF-β signaling are important in normal development and are commonly involved in body size control. For example, mutations in *dbl-1*, a TGF-β homologous genes, have a small body phenotype (Sma), whereas mutations in *lon-2*, a heparan sulfate proteoglycan, have a long body phenotype (Lon) [10,11]. Binding of LON-2 to DBL-1 interferes with the binding of DBL-1 to TβR and antagonizes TGF-β signaling in a dose-dependent manner [12].

TβR internalization is one of the pathways involved in the negative regulation of TGF-β signaling. TβR is internalized through the clathrin-coated pit pathway and the caveolin-coated caveolae pathway. TβR internalization through caveolae is known to downregulate TGF-β signaling by increasing the degradation of TβRI through lysosomes and proteasomes [13,14,15]. In contrast, Smad-dependent TGF-β signaling is internalized through clathrin-coated pits, and internalized TβR is recycled dependent on RAB-11 [16,17]. TβRI directly binds to the scaffolding domain of caveolin-1 and the β2 adaptin of the clathrin-associated protein (AP) complex [18,19].

We previously reported that *tyrosyl protein sulfotransferase-1* (*tpst-1*) and *suppressor-of-rolling-1* (*suro-1*) are required for normal cuticle formation in *C. elegans* [20,21]. In this manuscript, we identify the *suro-9* mutant to understand the molecular mechanism of cuticle formation. The *suro-9* mutant has a mutation in the *dpy-23* gene encoding the μ adaptin of the AP-2 complex, and AP-2 functions to maintain TGF-β signaling by inhibiting *cav-1* expression.

## 2. Results

### 2.1. DPY-23/AP2M1 is Required for the Normal Cuticle

**Figure 1:**
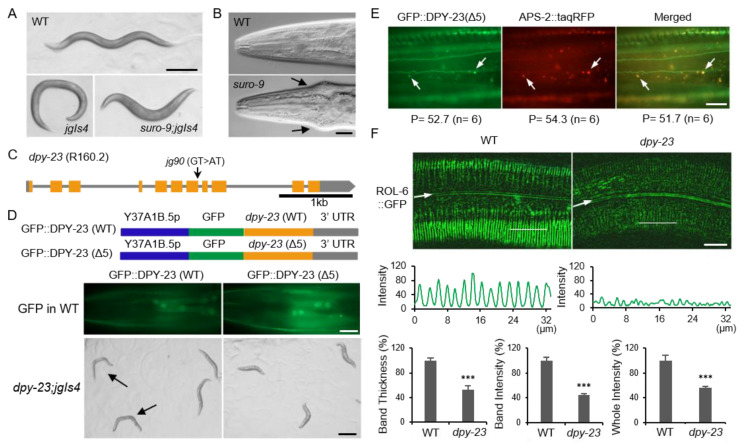
DPY-23/AP2M1 functions in normal cuticle formation. (**A**) The wild-type (WT), *jgIs4* and *suro-9; jgIs4* strains were observed using a dissecting microscope. The *suppressor-of-rolling-9* (*suro-9*) is one mutant among cuticle mutants that suppresses the rolling (Rol) phenotype of the *jgIs4* strain expressing the mutant ROL-6 collagen. Scale bars = 200 μm. (**B**) The head region of the *suro-9* mutant was compared to the wild type using a differential interference contrast (DIC) microscope. Arrows indicate the Jowls phenotype. Scale bar = 20 µm. (**C**) The genomic structure of the *dpy-23* gene. The arrow indicates the *suro-9 (jg90)* mutation. (**D**) Rescue experiments were performed with two different constructs expressing the wild-type DPY-23 (GFP::DPY-23 (WT)) or the mutant DPY-23 (GFP::DPY-23 (Δ5)) by the Y37A1B.5 promoter (Y37A1B.5*p*) (upper panel). Y37A1B.5 is expressed in the hypodermis and DPY-23 (Δ5) indicates the 5-amino acids-deletion form of DPY-23 derived from *suro-9 (jg90).* The transgenic nematode expressing either GFP::DPY-23 (WT) (left panel) or GFP::DPY-23 (Δ5) (right panel) in the wild-type background was observed using a fluorescence microscope (middle panel). The *dpy-23; jgIs4* strain expressing either GFP::DPY-23 (WT) (left panel) or GFP::DPY-23 (Δ5) (right panel) was observed using a dissecting microscope (bottom panel). Arrows indicate rolling animals. Scale bars = 20 μm (middle panel) and 500 μm (bottom panel). (**E**) The transgenic nematode expressing GFP::DPY-23 (Δ5) and APS-2::tagRFP simultaneously in the hypodermis was observed to examine the complex formation of the mutant DPY-23 protein with other subunits of the clathrin-associated complex II (AP-2). Arrows indicate AP-2 punctae. P = average number of punctae (n = worm number observed). P of the merged image indicates the number of overlapping punctae. Scale bar = 20 μm. (**F**) The expression of ROL-6::GFP in the *dpy-23* mutant was compared to the wild type. The fluorescence intensity was measured along the thin white line in the upper panel and was presented graphically in the middle panel. The relative band thickness, relative band intensity, and relative whole intensity of ROL-6::GFP in the wild type and the *dpy-23* mutant were quantified (bottom panel). Arrows indicate alae. Scale bar = 20 μm. Error bar = SEM. *** *p* < 0.001 versus WT.

The *suro-9* mutant is one of the cuticle mutants that suppress the rolling (Rol) phenotype of the *jgIs4* strain. The *jgIs4* strain expresses the mutant ROL-6 collagen protein derived from the *rol-6 (su1006)* mutant and has twisted cuticles [21]. The reason for using *jgIs4* without using the *rol-6* mutant was to exclude the intragenic suppressor and to find the extragenic suppressor. The *jgIs4* strain has a transgenic marker green fluorescent protein (GFP) expressed in the intestine and the hypodermis by the *tpst-1* promoter and exhibits a 100% Rol phenotype. The *suro-9* mutant displayed a pleiotropic phenotype and completely inhibited Rol (Figure 1A). Another feature of the *suro-9* mutant is a Jowls phenotype, which swells on both sides of the cuticle around the pharynx (Figure 1B). The Jowls phenotype is known to be closely related to clathrin-dependent endocytosis (CDE) [22]. Whole genome sequencing of *suro-9 (jg90)* revealed a mutation in the splicing donor site next to exon 7 in the *dpy-23* gene (Figure 1C). We sequenced a complementary DNA (cDNA) clone of the *dpy-23 (jg90)* mutant to investigate how splicing occurs in the *dpy-23* gene. As a result, it is expected that cryptic splicing will occur and a mutant DPY-23 protein with a 5-amino acids-deletion will be generated in the *dpy-23 (jg90)* mutant (Figure S1). DPY-23 is the orthologue of mammalian AP2M1, a μ adaptin of the AP-2 complex. The AP complex is a heterotetrameric protein complex, and its subunit is called an adaptin. Due to the complex terminology of adaptin, the nomenclature for *C. elegans* and human AP-2 is summarized in the table for better understanding (Table S1) [23].

Since most cuticular collagens are produced and secreted in the hypodermis, rescue experiments were performed to investigate whether DPY-23 function in the hypodermis is critical for cuticle formation. Two different GFP-fused DPY-23 (GFP::DPY-23) expression constructs driven by the Y37A1B.5 promoter (Y37A1B.5*p*) were generated. Both wild-type and mutant GFP::DPY-23 (WT and Δ5) proteins were expressed in the hypodermis (Figure 1D) [24]. The *dpy-23; jgIs4* strain expressing GFP::DPY-23 (WT) restored Rol and normal body length, but GFP::DPY-23 (Δ5) failed to rescue the *dpy-23* mutant (Figure 1D). Next, we investigated the ability of GFP::DPY-23 (Δ5) to form AP-2 complexes in vivo. The tetrameric AP-2 complex is known to consist of α, β2, μ2, and σ2 adaptins, while APS-2 is the σ adaptin of AP-2 in *C. elegans*. Since 98.2% of the GFP::DPY-23 (Δ5) puncta was localized at the same puncta as APS-2::tagRFP, the mutant DPY-23 could participate in the AP-2 complex (Figure 1E).

To investigate the association between Rol suppression and cuticle structure change, a ROL-6 collagen marker (ROL-6::GFP) was observed in the *dpy-23* mutant. ROL-6::GFP appeared in a regular stripe pattern in the wild-type cuticle, but in an irregular pattern in the *dpy-23* mutant. ROL-6::GFP intensity was significantly reduced in the *dpy-23* mutant compared to the wild type. The thickness of the ROL-6::GFP band was also reduced in the *dpy-23* mutant (Figure 1F).

### 2.2. The Lon-2 Mutation Suppressed the Dpy-23 Mutant Phenotype

**Figure 2:**
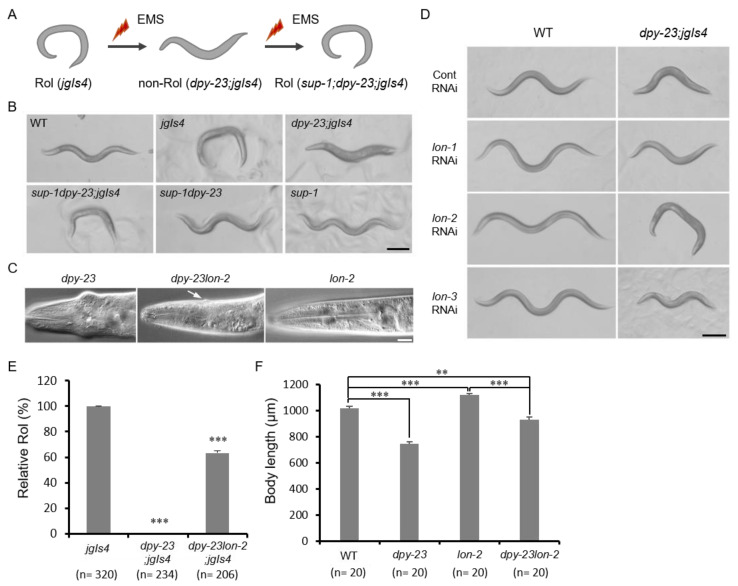
Suppression of the *dpy-23* mutant by the *lon-2* mutation. (**A**) The screening procedure of Rol and *dpy-23* suppressors using ethyl methane sulfonate (EMS). The *suppressor-1* (*sup-1*) mutant recovers Rol. (**B**) The *jgIs4, dpy-23; jgIs4, sup-1dpy-23; jgIs4, sup-1dpy-23 and sup-1* mutant strains were observed and compared to the wild type under the dissecting microscope. Scale bar = 200 μm. (**C**) The head regions of *dpy-23, dpy-23lon-2* and *lon-2* were compared. The arrow indicates the reminiscence of the Jowls phenotype. Scale bar = 20 μm. (**D**) The wild-type and *dpy-23; jgIs4* strains were treated with each RNA interference (RNAi) for the indicated *lon* genes and control (Cont) to examine *dpy-23* suppression. Scale bar = 200 μm. (**E**) Rol ratios of the *jgIs4, dpy-23; jgIs4* and *dpy-23lon-2; jgIs4* strains were compared. Error bar = SEM. *** *p* < 0.001 versus *jgIs4*. (**F**) Body lengths of the wild-type, *dpy-23, dpy-23lon-2* and *lon-2* strains were measured. Error bar = SEM. ** *p* < 0.01 and *** *p* < 0.001. These experiments were repeated more than three times and each result exhibited the same tendency.

We performed a suppressor screening to find the genetic interactors of *dpy-23*. As the *dpy-23* mutant suppressed the Rol phenotype of *jgIs4*, the *dpy-23; jgIs4* strain was mutagenized and rolling worms were selected as a suppressor (*sup*) mutant from the F2 population (Figure 2A). The tentatively named *sup-1* mutant was linked to *dpy-23* and the single *sup-1* mutant exhibited a Lon phenotype (Figure 2B). Because the *lon-2* locus is located near the *dpy-23* locus, the *lon-2* genomic coding region of the suppressor mutant was sequenced. As a result, *sup-1* showed a 4 bp deletion in exon 4 of the *lon-2* gene, and this mutation resulted in premature stop (Figure S2A). The short LON-2 protein with 186 amino acids expected to be made from the *lon-2 (jg108)* mutant would lose two functional domains, including RGD and SGSG, which are required to inhibit TGF-β signaling (Figure S2B) [25]. The Jowls phenotype of *dpy-23* was also suppressed by the *lon-2* mutation (Figure 2C).

Since *lon-2* suppressed *dpy-23*, we tested whether other *lon* genes were involved in the same process. Knockdown of three *lon* genes by RNA interference (RNAi) resulted in a Lon phenotype in the wild-type background, whereas only *lon-2* RNAi resulted in the recovery of Rol in the *dpy-23; jgIs4* strain in contrast to *lon-1* and *lon-3* (Figure 2D). Since the body length and Jowls phenotype of *dpy-23* appeared to be partially suppressed by *lon-2*, Rol ratios and body lengths of these mutants, including the *dpy-23* and *dpy-23lon-2* mutants, were measured. Rol ratio of *dpy-23; jgIs4* increased from 0% to 65% by the *lon-2* mutation (Figure 2E). The body length of the *dpy-23lon-2* mutant was between the *dpy-23* mutant and the *lon-2* mutant (Figure 2F). Knockdown of *lon-1* and *lon-3* significantly increased the body length of *dpy-23; jgIs4* but did not restore the Rol phenotype (Figure S3).

The genetic interaction of ECM-associated genes including *dpy-23* and a BMP-like DBL-1 is already known [26]. A glypican protein LON-2 is known as a negative regulator of the TGF-β signaling pathway by sequestering DBL-1, and the TGF-β signal is known as a major regulatory signal for body size [12,25]. TβRI is also known to interact with AP-2 complex by direct binding to β2 [18]. Taken together, the reduced TGF-β signal in the *dpy-23* mutant could be compensated by the *lon-2* mutation. These results suggest that the wild-type cuticle and body length depend on the balance of TGF-β signaling, which is positively regulated by DPY-23 and negatively regulated by LON-2.

### 2.3. Caveolin Is Upregulated in the Dpy-23 Mutant

Subsequently, RNA-seq analysis was performed using wild-type and *dpy-23* mutant RNAs to find genes whose expression is changed by the *dpy-23* mutation. Overall, the change in the expression of ECM-related genes, including collagens, was the largest (Figure S4). These results are consistent with the abnormal cuticle phenotype of the *dpy-23* mutant. Among the genes showing significant changes in expression, *cav-1,* an orthologue of *caveolin-1*, was remarkable except for the nematode specific gene. Caveolin-1 is a key protein in caveolae responsible for clathrin-independent endocytosis (CIE) [27]. While CDE has been shown to promote TGF-β-induced Smad activation and transcriptional responses, lipid rafts/caveolae are indicated to facilitate the degradation of TβRs [15]. There are two caveolin genes in *C. elegans*, *cav-1* and *cav-2* [28,29]. In contrast to *cav-1*, *cav-2* expression slightly changed in the *dpy-23* mutant compared to the wild type (Table S2).

**Figure 3:**
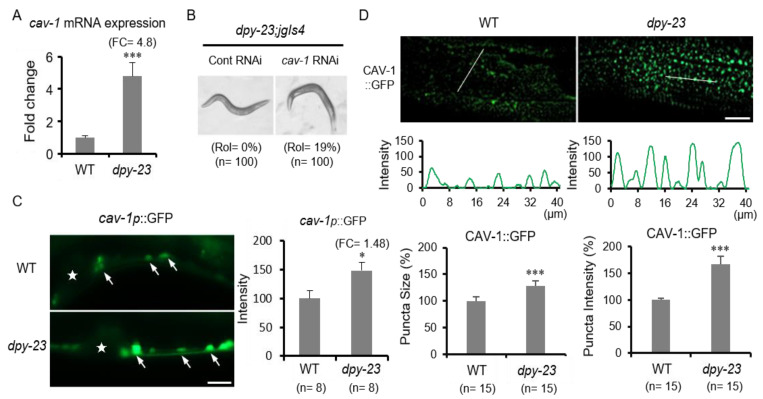
Increase of *cav-1* expression in the *dpy-23* mutant. (**A**) The mRNA expression of *cav-1* gene in the *dpy-23* mutant was compared to the wild type using a quantitative reverse transcription PCR (qRT-PCR). Error bar = SD. FC = fold change. (**B**) The *dpy-23; jgIs4* strain was treated with *cav-1* RNAi to investigate its effect on cuticle formation. (**C**) The GFP reporter expressed by the *cav-1* promoter (*cav-1p*::GFP) was observed in wild type and *dpy-23* to investigate *cav-1* transcription in vivo. Because *cav-1p*::GFP expression was more visible in neurons than in other tissues, *cav-1p*::GFP expression in ventral neurons was compared. Asterisks indicate vulvae, and arrows indicate the cell bodies of ventral neurons. Scale bar = 20 μm. The measured GFP intensity is shown in the graph on the right. Error bar = SEM. (**D**) CAV-1::GFP expressed in the hypodermis by Y37A1B.5*p* was observed and compared (upper panel). CAV-1::GFP intensity was measured along thin white lines in wild type and *dpy-23* (middle panel). The measured puncta size and intensity of CAV-1::GFP are shown in the graph (bottom panel). Scale bar = 20 μm. Error bar = SEM. * *p* < 0.05 and *** *p* < 0.001 versus WT.

To verify RNA-seq results, the quantitative reverse transcription PCR (qRT-PCR) of *cav-1* was performed using total RNAs from the wild-type and *dpy-23* mutant strains. In the *dpy-23* mutant, *cav-1* mRNA increased up to 4.8 times compared to the wild type (Figure 3A). We also performed *cav-1* RNAi in *dpy-23; jgIs4* to investigate whether the increase in *cav-1* by the *dpy-23* mutation affects cuticle formation. The results showed that *dpy-23; jgIs4* partially recovered the Rol phenotype from 0% to 19% by *cav-1* knockdown (Figure 3B). Next, we examined *cav-1* expression in vivo using a GFP reporter expressed by the *cav-1* promoter (*cav-1p*::GFP) and the full-length CAV-1-GFP fusion reporter (CAV-1::GFP) expressed in the hypodermis by Y37A1B.5*p*. The *cav-1p*::GFP was observed in ventral neurons, and the expression level of *cav-1p*::GFP in the *dpy-23* mutant was significantly increased compared to the wild type (Figure 3C). CAV-1::GFPs were expressed as punctae near the hypodermal cell membrane, possibly representing individual caveolae or small caveolar vesicles [30]. In the *dpy-23* mutant, the size and number of CAV-1::GFP punctae increased compared to the wild type, and the intensity of CAV-1::GFP also increased (Figure 3D). These results suggest that both mRNA and protein levels of *cav-1* were increased in the *dpy-23* mutant.

### 2.4. TGF-β Signaling Was Reduced in the Dpy-23 Mutant

**Figure 4:**
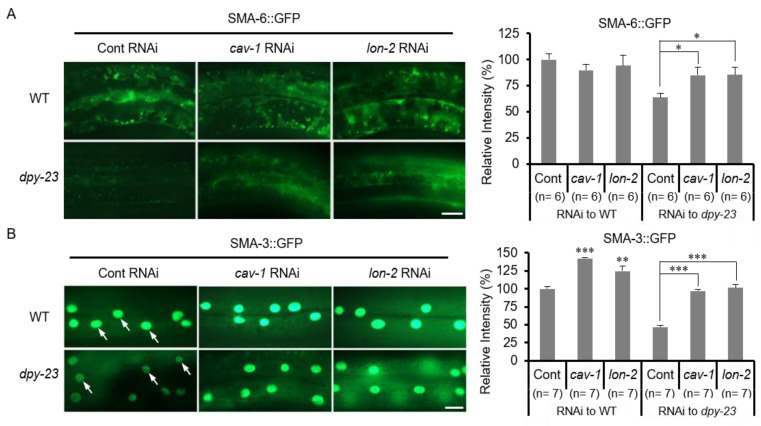
TGF-β signaling in the *dpy-23* mutant. The expression level of SMA-6/TGF-β receptor I (TβRI) and SMA-3/R-Smad in the *dpy-23* mutant compared to the wild type using SMA-6::GFP and SMA-3::GFP expressed in the hypodermis by Y37A1B.5*p*. The transgenic worms expressing SMA-6::GFP (**A**) and SMA-3::GFP (**B**) in the wild type and the *dpy-23* mutant were treated with *cav-1* and *lon-2* RNAi to examine the change of TGF-β signals. The fluorescence intensity of SMA-6::GFP and SMA-3::GFP was measured and shown graphically in the right panel of each figure. In case of SMA-3::GFP quantification, nuclear localized SMA-3::GFP was measured in contrast to SMA-6::GFP, which is measured with total GFP. Arrows indicate nuclei of hypodermal cells. Scale bar = 10 μm. Error bar = SEM. * *p* < 0.05, ** *p* < 0.01 and *** *p* < 0.001 versus Cont.

Since *lon-2* and *cav-1* knockdown suppressed the phenotype of the *dpy-23* mutant, TGF-β signaling may be altered in the *dpy-23* mutant. To investigate the change of the TGF-β signal in the *dpy-23* mutant, the expression of SMA-6 and SMA-3 was observed using a transgenic strain expressing SMA-6::GFP and SMA-3::GFP, respectively. SMA was named because this mutation showed a small phenotype [31]. SMA-6 is a TβRI homologue and SMA-3 is an R-Smad homologue of *C. elegans* [32]. SMA-6::GFPs expressed by Y37A1B.5*p* were decreased in the *dpy-23* mutant compared to the wild type (Figure 4A). The total expression and nuclear translocation of SMA-3::GFPs expressed in the hypodermis by Y37A1B.5*p* were also reduced in the *dpy-23* mutant (Figure 4B). These results suggest that DPY-23 is required to maintain TGF-β signaling to form normal cuticles and to maintain SMA-6/TβRI and SMA-3/R-Smad at the protein level.

Next, we investigated whether the reduced TGF-β signal in the *dpy-23* mutant could be recovered by *cav-1* and *lon-2* knockdown. For this purpose, *dpy-23* mutants expressing SMA-6::GFP and SMA-3::GFP, respectively, were treated with *cav-1* or *lon-2* RNAi. In the wild-type background, SMA-6::GFP was not affected by *cav-1* or *lon-2* knockdown, whereas in the *dpy-23* mutant background, weak SMA-6::GFP was increased by *cav-1* or *lon-2* knockdown (Figure 4A). In contrast, SMA-3::GFP was increased by *cav-1* or *lon-2* knockdown in both the wild type and the *dpy-23* mutant (Figure 4B). These results suggest that DPY-23, CAV-1 and LON-2 act in regulating the TGF-β signaling pathway, and that DPY-23 functions are opposite to CAV-1 and LON-2.

### 2.5. DPY-23 is Required for the Expression of Several Collagen Genes

**Figure 5:**
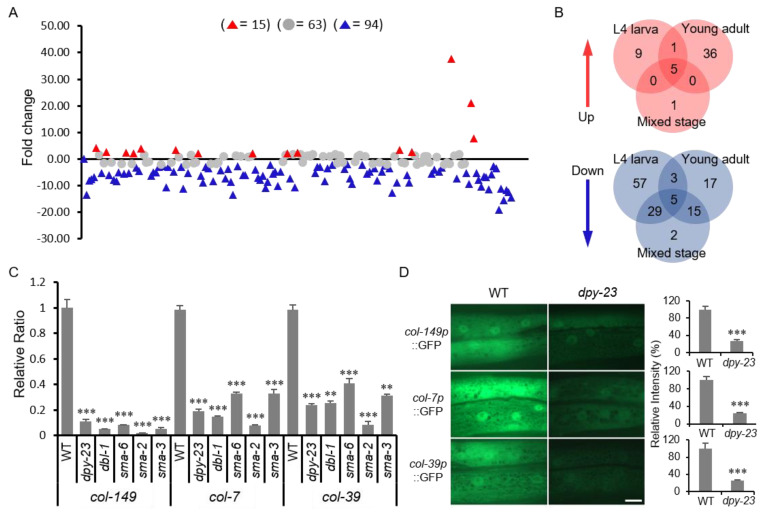
Expression of collagen genes in the *dpy-23* mutant. (**A**) The changes in the expression of 170 collagen genes in the *dpy-23* mutant compared to wild type according to the RNA-seq results of L4 larvae are shown in the graph. Gray circles indicate collagen with a difference in expression fold change less than 2 (FC < 2), and triangles indicate that FC is more than 2 (FC > 2). Red color indicates an increase in the *dpy-23* mutant, and blue indicates a decrease. The x-axis is a listing of 170 collagen genes. (**B**) The number of significantly changed collagen genes (FC > 2) in the three RNA-seq results is shown as a diagram. (**C**) The mRNA expression of three collagen genes including *col-7*, *col-39* and *col-149* was measured by qRT-PCR in the *dpy-23* and TGF-β signaling mutants. Error bar = SD. (**D**) The GFP reporter expressed by each collagen promoter was observed to compare *dpy-23* with wild type. The measured GFP intensity was shown in graphs (right panel). Scale bar = 10 μm. Error bar = SEM. ** *p* < 0.01 and *** *p* < 0.001 versus WT.

Since the major phenotype of the *dpy-23* mutant is an abnormality in the cuticle, 170 collagen genes were extracted from RNA-seq results and analyzed. We performed RNA-seq analysis using total RNAs extracted from L4, young adult and mixed-stage wild-type and *dpy-23* worms, respectively. In the *dpy-23* mutant, the number of collagens with fold change (FC) greater than 2 (FC > 2) surpassed the number of collagens with FC less than 2 (FC < 2). In particular, the number of collagens with reduced expression was much greater than the number of collagens with increased expression (Figure 5A). In RNA-seq results, the number of commonly increased or decreased collagens was only 5 in all 3 cases, and the *dpy-23* mutation had the greatest effect on collagen expression during L4 larvae (Figure 5B). Compared to the wild type, the expression of *rol-6* gene in the *dpy-23* mutant decreased significantly in the L4 stage (FC = −11.2), but slightly increased in adults (FC = 2.39) (Table S2). The *rol-6* gene is mainly expressed in L2, L3 and L4 larvae in the fragment per kilobase of transcript per million mapped reads (FPKM) expression data arranged in Wormbase (http://wormbase.org). Therefore, we used qRT-PCR to investigate the expression of *rol-6* in L4 larvae. As a result, the expression of *rol-6* was decreased in *dpy-23, dbl-1, sma-2*, and *sma-6* mutants compared to the wild type (Figure S5). SMA-2 is a homologue of human R-Smad [11]. These results suggest that the reduction of *rol-6* in the *dpy-23* mutant is associated with the Rol suppression phenotype.

To verify the RNA-seq results, the expression of three collagen genes in the *dpy-23* mutant was examined by qRT-PCR. The *col-149*, *col-7* and *col-39* genes were selected from the five collagens that were decreased at all stages of the *dpy-23* mutant (Table S2). All three collagen mRNAs were drastically reduced in the *dpy-23* mutant compared to the wild type (Figure 5C). The mRNA expression levels of these collagen genes were also investigated in *dbl-1*, *sma-6*, *sma-2* and *sma-3* mutants. In these mutants, mRNA expression of three collagen genes decreased similarly to the *dpy-23* mutant (Figure 5C). We generated a transgenic strain expressing a GFP reporter using the promoter of each collagen gene. The transgenic strain was crossed with the *dpy-23* mutant and compared to GFP expression level in the wild type. As a result, the expression of *col-149p*::GFP, *col-7p*::GFP and *col-39p*::GFP was significantly reduced in the *dpy-23* mutant compared to the wild type (Figure 5D). These results suggest that the transcription of several collagens is dependent on DPY-23 and TGF-β signals.

## 3. Discussion

*C. elegans* cuticle is a good in vivo model for studies of fibrosis and ECM remodeling due to active ECM production. In this study, we found that DPY-23/AP2M1 maintains the TGF-β signal that regulates collagen expression and cuticle formation at the organism level. The *dpy-23* mutant exhibits a variety of phenotypes like other µ adaptin mutants. The most representative phenotype of *dpy-23* is dumpy, but in *C. elegans*, the function of AP-2 has mainly been studied in neurons. In this study, we identified the functional and molecular mechanisms of AP-2 in cuticle formation.

As a result of the suppressor mutant screening of *dpy-23*, the *lon-2* mutant appeared, and the TGF-β pathway was found to be involved in the formation of an abnormal cuticle in the *dpy-23* mutant. The TGF-β signaling pathway is important for ECM production, and is a major signal for the formation of tissue microenvironments, especially in human cancers and fibrotic diseases [33]. LON-2 is known as a negative regulator of the TGF-β signaling pathway by sequestering DBL-1, and the TGF-β signal is known as a major regulatory signal for body size in *C. elegans* [12,25]. In addition, increased expression of *cav-1* reduced TβRI, and *cav-1* RNAi resulted in suppression of *dpy-23*. These results suggest that cuticle abnormality in *dpy-23* is associated with a decrease in TGF-β signaling.

One of the typical signaling pathways regulated by AP-2 is the Wnt pathway. AP-2 regulates the recycling of MIG-14/Wntless in neurons, and neuronal cell migration is affected by the *dpy-23* mutation [34]. LON-2 is known to regulate the movement of neurons through genetic interaction with EGL-20/Wnt [35]. Thus, we investigated whether the Wnt pathway is involved in cuticle formation. We observed a recycling problem with MIG-14::GFP by *dpy-23* RNAi in the *C. elegans* intestine (Figure S6A). However, the Rol phenotype of *jgIs4* was not affected by *mig-14* knockdown, and the ROL-6::GFP expression pattern and cuticle structure in the *mig-14* mutant was similar to that of the wild type (Figure S6B,C). Therefore, AP-2 function is related to TGF-β signaling rather than the Wnt signal in the hypodermis that produces the cuticle.

SMA-6::GFP is increased by AP-2 knockdown in contrast to DAF-4/TβRII in *C. elegans* intestine [36]. The increase of SMA-6::GFP in the intestine by AP-2 knockdown is similar to that of MIG-14::GFP (Figure S6A). In contrast, in this work, SMA-6::GFP decreased in the hypodermis of the *dpy-23* mutant. This difference is estimated as the difference in expression of CAV-1 depending on tissues. To better understand the regulation of TβRI in *C. elegans* hypodermis, transgenic worms that simultaneously express SMA-6::GFP and APS-2::tagRFP or CAV-1::tagRFP were generated. Overexpression of CAV-1::tagRFP significantly reduced the expression of SMA-6::GFP in contrast to overexpression of APS-2::tagRFP (Figure S7A). Overexpression of SMA-6::GFP appeared to increase the body length of *C. elegans*. However, this effect of SMA-6::GFP overexpression was eliminated by CAV-1::tagRFP overexpression, so the body length of the transgenic strain simultaneously expressing SMA-6::GFP and CAV-1::tagRFP did not increase (Figure S7B). These results did not show that AP-2 and CAV-1 directly regulate SMA-6 by endocytosis, but strongly suggest that AP-2 and CAV-1 are oppositely involved in the quantitative regulation of SMA-6 in the plasma membrane of hypodermal cells.

As a result of RNA-seq, the most changed gene cluster in the *dpy-23* mutant involved collagens and ECM-related genes. We have not investigated which collagen genes are direct targets of TGF-β signaling as some collagens have already been reported as direct target genes for TGF-β signaling in *C. elegans* by ChIP-seq analysis of SMA-3 [37]. However, we showed that the expression of *col-149, col-7,* and *col-39* genes decreased drastically in the *dpy-23* mutant as well as in the TGF-β-pathway-related mutants (Figure 5C,D). The small number of commonly changed collagens in all three RNA-seq cases suggests that collagen expression is regulated differently according to TGF-β signals depending on developmental stages of *C. elegans*. In fact, it has been reported that *col-41* and *rol-6* are promoted during L2 stage by DBL-1 and Smad signals but repressed in adults, and *dpy-2* and *dpy-9* mutations have stage-specific effects on DBL-1 signaling [37,38]. Regarding the Rol phenotype, decreased expression of *rol-6* could be a direct cause of the *dpy-23* mutant phenotype. Likewise, a decrease in the expression of *rol-6* is also observed in TGF-β pathway mutants, so that AP-2 and TGF-β signaling have a large common part in cuticle formation.

**Figure 6:**
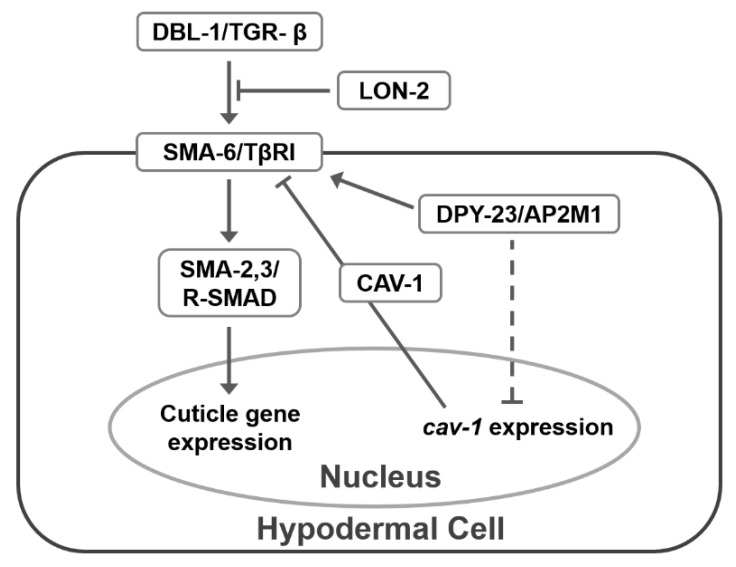
Model for DPY-23, LON-2 and CAV-1 function in TGF-β signaling. In the hypodermis of *C. elegans*, TGF-β signaling promotes collagen expression, which is required for normal cuticle formation. Inhibition of TGF-β signaling could result in a decreased cuticle gene expression. Outside of the cell, LON-2 binding to TGF-β antagonizes TGF-β signaling. Alternatively, inside of the cell, increased CAV-1 by the *dpy-23* mutation could decrease TGF-β signaling through TβRI downregulation.

We propose a model for cuticle formation involving the regulation of TGF-β signaling by AP-2 in the hypodermis of *C. elegans,* as shown in Figure 6. The AP-2 complex is required for the TGF-β signal transduction and the activated TGF-β signal seems to inhibit *cav-1* gene expression under normal conditions [14]. However, the increased expression of *cav-1* at both mRNA and protein levels leads to a decrease of TβRI in the *dpy-23* mutant. Increased CAV-1 can increase the degradation of TβRI in human cells via lysosomes or proteasomes [15,39]. A reduction in TGF-β signaling decreases the expression of cuticle-related and ECM-associated genes. LON-2 also inhibits TGF-β outside of the cell, thereby reducing TGF-β signaling, which counteracts DPY-23.

This study demonstrated that the maintenance of TGF-β signaling, a major signal for regulating ECM production, is dependent on AP-2, and that the molecular mechanism underlying this regulation involves caveolin-1 expression in the *C. elegans* cuticle formation. These findings can provide a clue to understanding the molecular mechanism of the complex process of ECM regulation.

## 4. Materials and Methods

### 4.1. C. elegans Culture and Strains

The standard reference strain N2 was used as the wild-type strain, and all transgenic strains were generated by injecting transgenes in the N2 background. The wild-type and other strains were fed on OP50 *Escherichia coli* growing on the standard nematode growth media (NGM) plate at 20 °C as described by Dr. Brenner [40]. The *C. elegans* strains used in this study were *cav-1 (ok2080)*, *dbl-1 (wk70), dpy-23 (jg90), jgIs4 [pRF4 (rol-6d), tpst-1p::gfp], jgIs5 [rol-6::gfp, ttx-3::gfp], lon-2 (jg108)*, *sma-2 (e502)*, *sma-3 (wk30)* and *sma-6 (e1482)*. The *jgIs4* strain expresses mutant ROL-6 collagens originated from the *rol-6 (su1006)* mutant, and *jgIs5* expresses ROL-6::GFP. 

### 4.2. Mutagenesis and Mutant Cloning

The *suro-9* mutant was out-crossed 9 times with wild type, and whole genome sequencing of *suro-9* revealed a mutation in R160.1. The ORF R160.1 encodes the *dpy-23* gene. The *suro-9 (jg90)* mutant was segregated from *suro-9 (jg90); jgIs4* by crossing with wild type. Whole genome sequencing of *suro-9 (jg90)* was performed by Macrogen (Seoul, Korea) and the genomic data were analyzed using MAQGene [41]. Finally, R160.1 cDNA amplified by PCR from the *dpy-23* mutant RNA as a template was sequenced to verify the mutation. To find genetic interactors of *dpy-23*, the *dpy-23*; *jgIs4* strain was mutagenized using ethyl methane sulfonate (EMS, Sigma–Aldrich Chemicals, St. Louis, MO, USA). L4 worms were harvested and treated with EMS (final concentration 0.05 M) at 20 °C for 4 hours. P0 worms were recovered on ten 90 mm NGM plates and kept until starved. We chunked F2 worms to 100 new 60 mm NGM plates and chose suppressor mutants after 2 days. Finally, 18 rolling mutants were selected. Because the suppressor mutant was hardly segregated from the *dpy-23* mutant during out-crossing, the suppressor was predicted to be closely located with the *dpy-23* locus on the same X chromosome. In addition, the single suppressor mutant separated from *dpy-23* displayed a Lon phenotype, and *lon-2* RNAi also suppressed *dpy-23; jgIs4* (Figure 2B). Based on these results, we performed sequencing of the *lon-2* genomic region amplified by PCR using the genomic DNA from 18 suppressor mutants. Fourteen mutants had the same mutation in the *lon-2* gene (Figure S2), but the other 4 mutants had no mutations in the *lon-2* gene.

### 4.3. Fluorescent Protein Fusion Reporter Constructs and Microinjection

We used pPD95.77 and pPD117.01 Fire vectors (A. Fire, Stanford University, Stanford, CA, USA) as GFP vectors, and pIJ559 as a tagRFP vector (Dr. S. Lee, KAIST, Daejeon, Korea). We used the 1.2 kb 5′ upstream region of the Y37A1B.5 gene to express proteins in the hypodermis, and pJG452 was constructed by cloning the Y37A1B.5 promoter into pPD95.77 using HindIII and PstI sites. The primers used for the amplification of the Y37A1B.5 promoter region were Y37A1B.5-03 (5′ TAT AAG CTT AGC GAG TGA CAT TTG CCG 3′) and Y37A1b.5-02 (5′ TAT CTG CAG TTT GGT TTT TGG GAT TTT TGA TCT GC 3′). We observed a GFP expression in the hypodermis of the transgenic strain having pJG452. The final concentration of the microinjection mixture was 150 µg/mL, including injection markers such as 50 µg/mL pRF4 (*rol-6*) and 5 µg/mL pCFJ90 (*myo-2p*::mCherry) used in the wild type. When the *unc-119* mutant was used for the host strain, pDP#MM16B, which expresses the wild-type *unc-119* gene, was used for the injection marker. DPY-23-, and APS-2- fluorescent protein (FP) fusion plasmids were injected with a 20–50 µg/mL concentration and pBluescript SK+ was added as necessary to adjust the final DNA concentration. The reporter plasmids used in this study were pJG1136 (Y37A1B.5*p*::GFP::DPY-23 (WT)), pJG1137 (Y37A1B.5*p*::GFP::DPY-23 (Δ5)), pJG1243 (Y37A1B.5*p*::tagRFP::APS-2), pJG1349 (Y37A1B.5*p*::MIG-14::GFP), pJG1514 (Y37A1B.5*p*::SMA-3::GFP), pJG1515 (Y37A1B.5*p*::SMA-6::GFP), pJG1518 (Y37A1B.5*p*::GFP::CAV-1), pJG1520 (*cav-1p*::GFP), pJG1543 (Y37A1B.5*p*::tagRFP::CAV-1), pJG1567 (*col-149p*::GFP), pJG1568 (*col-39p*::GFP), and pJG1569 (*col-7p*::GFP).

### 4.4. RNA Extraction and RT-PCR

The wild type and the *dpy-23 (jg90)* mutant were cultured on four 60 mm diameter NGM plates respectively and were harvested before starvation for RNA preparation. After 4 volumes of trizol reagent (15596-026, Invitrogen, Carlsbad, CA, USA) was added to worm suspension, worm/trizol suspension was mixed well by vortex for 1 min and kept at 80 °C. The next day, 17% of the total volume of chloroform was added to the rapidly thawed worm/trizol suspension and mixed well by vortex. The general isopropanol precipitation method was followed, and RNA pellets were dissolved in 100 µL TE (pH 7.2) solution. Reverse transcription (RT) was performed using the iScript cDNA synthesis kit (170-8890, Bio-Rad, Hercules, CA, USA) according to the manufacturer’s protocol. 

### 4.5. RNA Interference (RNAi)

RNAi plasmids were constructed using cDNAs of *cav-1, lon-1, lon-2, lon-3* and *mig-14.* The amplified PCR product for each gene using RT-PCR was cloned into the L4440 vector, and RNAi plasmids were transformed into *E. coli* HT115. The bacteria having each RNAi construct were seeded onto NGM plates with 100 μg/mL ampicillin and 1 mM IPTG (isopropyl β-D-1-thiogalactoside) after 6 h incubation at 37 °C in a shaking incubator [42]. The L4 larva was transferred to the RNAi plate and F1 progeny was observed. 

### 4.6. RNA-seq Analysis

Total RNAs extracted from the wild type and *dpy-23* mutant at mixed, L4 and young adult stages were sent to Macrogen (Seoul, Korea) for the RNA seq analysis. We prepared and sent more than 1 µg of RNA (100 μg/mL or above) for each sample. The basic analysis of RNA-seq results was performed by Macrogen, and we selected *cav-1* and collagen genes among the markedly changed genes in expression.

### 4.7. Microscopy and Statistics

Fluorescent microscopy was performed using an Axio Imager A2 compound microscope with a 63× lens (Zeiss, Oberkochen, Germany). Worms were prepared in an M9 buffer containing 2.5 mM levamisole on the slide glass in order to observe and take pictures. The GFP expression or intensity was measured using the ImageJ program (https://imagej.nih.gov/ij/). We also used the Lionheart FX automated microscope to compare GFP expression by quantity. Acquired images by Lionheart FX on a 40× lens on the slide glass were analyzed fluorescent quantity using the Gen5 software provided by the manufacturer. Statistical analysis in this study, such as standard deviation (SD), standard error (SEM) and *t*-test were performed using Sigmaplot (Systat software). *P* values *, **, *** were defined as <0.05, <0.01, and <0.001 respectively.

### 4.8. Rol Ratio and Body Length Measurement

For counting Rol worms, five L4 larvae were transferred to NGM plate or RNAi plate and were removed after laying 50–100 eggs. When F1 progeny grew up to young adults, the number of total and Rol worms was counted under a dissecting microscope. To measure the body length of worms, the fifty L4 larvae were picked from NGM plates of the mixed stage group and were cultured at 20 °C for one day. Next day, these young adult worms were mounted on 2% agarose on the slide glass by fixing with 10 mM sodium azide and were taken pictures using Axio Imager M2 microscope (Zeiss). The body length of each worm was measured using the Image J from the photograph.

### 4.9. Quantitative Real-Time PCR (qRT-PCR)

After RNA extraction, reverse transcription was performed for cDNA synthesis using the iScript cDNA synthesis kit (170-8890, Bio-Rad). Then, quantitative real-time PCR was performed via the Roche Light Cycler^®^ 96 using FastStart Essential DNA Green Marter (Roche, Basel, Switzerland). All experiments were tested in triplicate, and the relative transcript abundance analyzed for each tested gene was normalized to the expression levels of the housekeeping gene, actin (*act-1*). Error bars in the qRT-PCR graph demonstrated the standard deviation (SD) of the mean of the triplicate measurements. 

## 5. Conclusions

In this study, we showed that the cuticle formation and collagen expression was affected by TGF-β signals regulated by LON-2, CAV-1 and DPY-23 at the organism level using *C. elegans* genetics. DPY-23/AP2M1, a µ adaptin of AP-2 functions in normal cuticle formation by maintaining the TGF-β signaling pathway. LON-2, a negative regulator of TGF-β antagonized DPY-23 function parallelly, and the increased CAV-1 downregulated the TGF-β signal in the *dpy-23* mutant. Decreased expression of many collagen genes, including *rol-6*, due to the attenuation of the TGF-β signal in the *dpy-23* mutant resulted in abnormal cuticles.

## Data Availability

The data presented in this study are openly available in (RNA-seq of *C. elegans* dpy-23) at (https://www.ncbi.nlm.nih.gov/bioproject/PRJNA699748), reference number (SAMN17808522: dpy-23 L4 (TaxID: 6239)]).

## Supplementary Materials

Supplementary materials can be found at https://www.mdpi.com/1422-0067/22/4/1639/s1.

## Abbreviations

AP-2	Clathrin-associated protein complex II
ECM	Extracellular matrix
Dpy	dumpy
Lon	Long
Sma	Small
TGF-β	Tumor growth factor-beta
TβR	TGF-β receptor
CDE	clathrin-dependent endocytosis
CIE	clathrin-independent endocytosis
qRT-PCR	quantitative reverse transcription PCR
