# Supplementary material for: AP2M1 Supports TGF-β Signals to Promote Collagen Expression by Inhibiting Caveolin Expression
Source: Int J Mol Sci. 2021 Feb 6;22(4):1639. doi: 10.3390/ijms22041639 (PMC7915421; doi:10.3390/ijms22041639)
Supplement: Supplementary file 1 [file ijms-22-01639-s001.pdf]

Supplementary table 1. AP-2 complex proteins in *C. elegans* and *H. sapiens*

Subunit	General name	*C. elegans*	*H. sapiens*
Large	α adaptin	APA-2	AP2A1, AP2A2
	β2 adaptin	APB-1 [a]	AP2B1
Medium	μ2 adaptin	DYP-23	AP2M1
Small	σ adaptin	APS-2	AP2S1

[a] APB-1 is the β adaptin of AP-1 and AP-2 complexes in *C. elegans*

Nomenclature for *C. elegans* and human AP-2 is summarized with reference [29].

Supplementary table 2. Fold change of RNA seq analysis in the *dpy-23* mutant compared to the wild type

	cav-1	*cav-2*	*rol-6*	*col-149*	*col-7*	*col-39*
Mixed stage	5.97	1.15	-1.89	-4.21	-4.82	-2.02
L4 larva	19.89	2.27	-11.20	-8.21	-3.58	-3.50
Young adult	12.35	1.85	2.39	-18.08	-2.82	-2.86

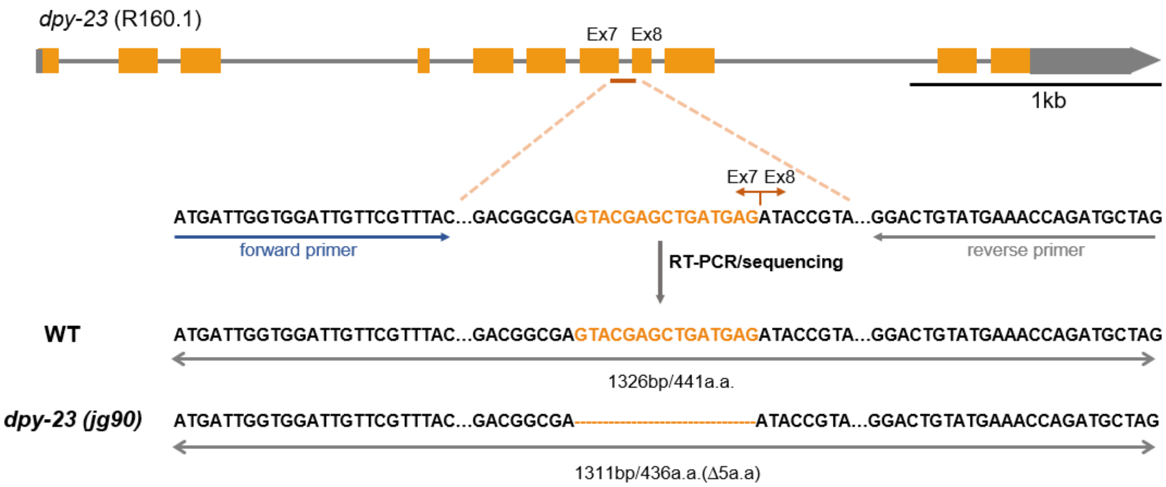

Figure S1. Splicing of *dpy-23* RNA in the *dpy-23 (jg90)* mutant. Since the whole genome sequencing and further conventional sequencing of *dpy-23* resulted in a mutation at the splicing donor site next to exon 7. Therefore, RT-PCR using the *dpy-23* RNA as a template and sequencing of *dpy-23* complementary DNA (cDNA) were performed to examine the splicing event in the *dpy-23* mutant. The cDNA sequences of wild type (WT) and *dpy-23 (jg90)* are compared. The sequencing result showed 15 bp deletion by the cryptic splicing. The *dpy-23* mutant is predicted to express the mutant DPY-23 proteins with 5 amino acids deletion. Ex7= exon 7 and Ex8= exon 8.

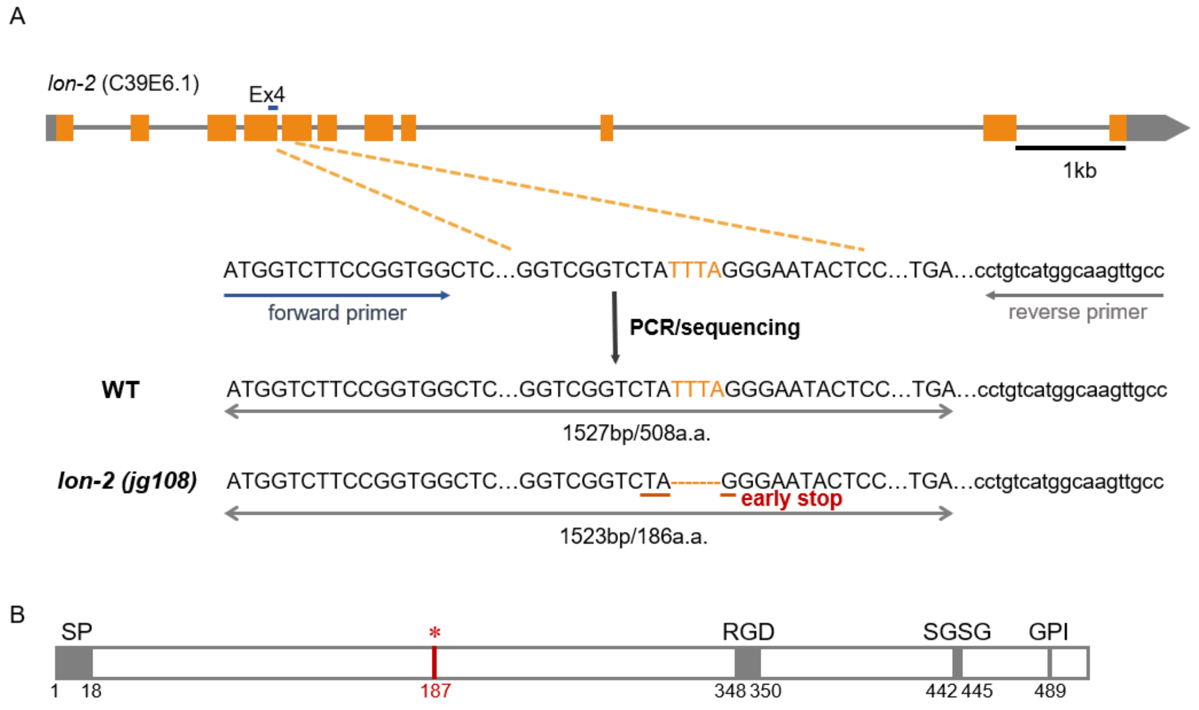

Figure S2. Identification of the *lon-2 (jg108)* mutation. (A) The genomic structure of *lon-2* and the sequencing result of *lon-2 (jg108)*. In order to find the correct mutation in the *lon-2* gene, the amplified PCR product was sequenced using the genomic DNA of the *lon-2* mutant as a template. There is 4 bp deletion in the exon 4 (Ex4) of the *lon-2* gene in the *lon-2* mutant, and this mutation is predicted to generate an early stop. (B) The protein structure of LON-2. The short LON-2 protein having 186 amino acids would be made in the *lon-2* mutant and is predicted to lose essential domains including RGD and SGSG domains. The asterisk indicates the stop site produced by the *lon-2 (jg108)* mutation.

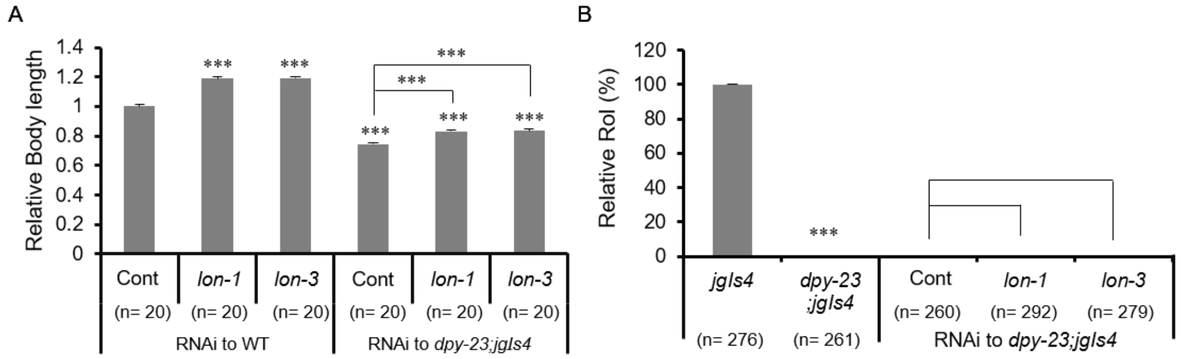

Figure S3. The body length and Rol ratio of the wild type and *dpy-23; jgIs4* strains. The wild type and the *dpy-23; jgIs4* mutant were treated with RNAi of control (Cont), *lon-1* and *lon-3*. (A) The young adult worms were taken pictures and their body length was measured using ImageJ. (B) The rolling worms were counted in the adult stage and each strain is indicated on the x-axis. Left two strains were fed on the normal NGM plate without any RNAi treatment. WT= wild type. Error bar= SEM. ***$P < 0.001$ versus Cont (A) and *jgIs4* (B).

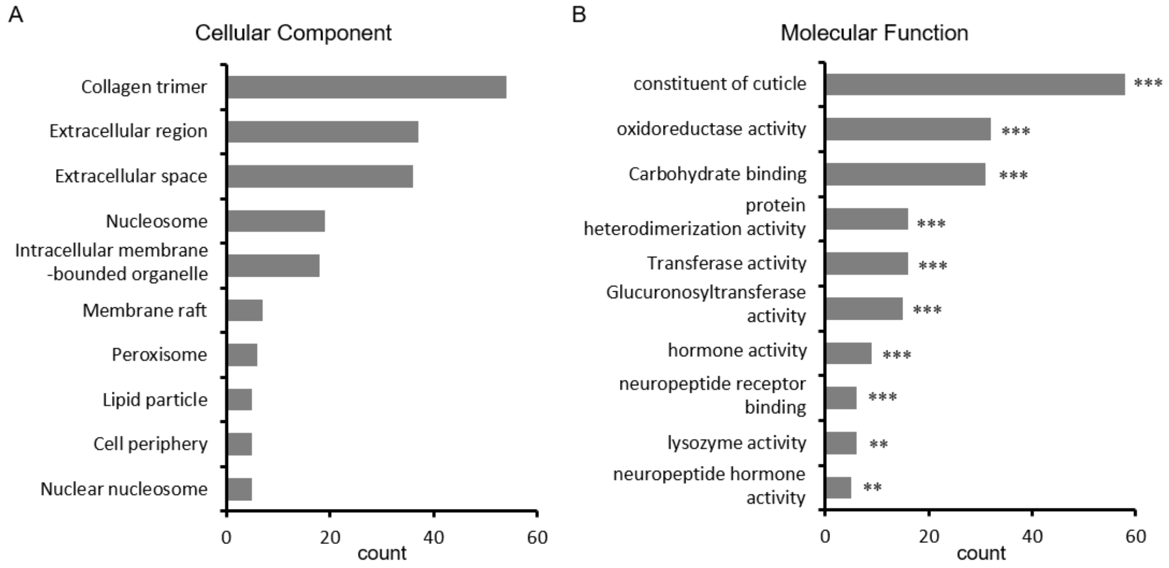

Figure S4. Results of the RNA seq analysis using the wild type and the *dpy-23* mutant. Genes whose expression was significantly changed in the *dpy-23* mutant were classified according to cellular components (A) and molecular functions (B). *$P < 0.05$, **$P < 0.01$ and ***$P < 0.001$ versus WT.

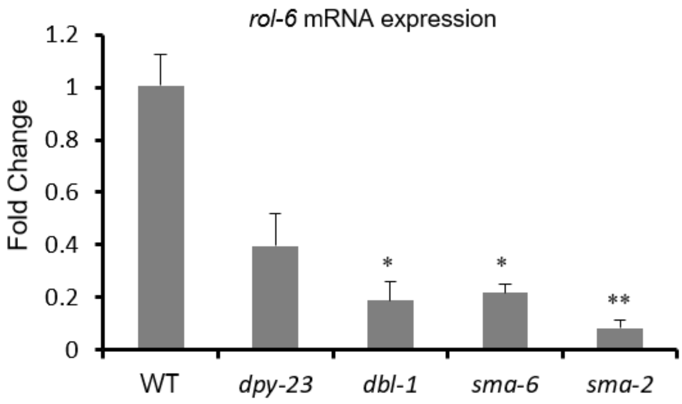

Figure S5. The expression level of the *rol-6* gene measured by qRT-PCR. RNAs extracted from the synchronized L4 larva of each strain indicated in the x-axis. The mRNA expression of *rol-6* was normalized by actin (*act-1*). Error bar= SD. **P*< 0.05 and ***P* < 0.01 versus WT.

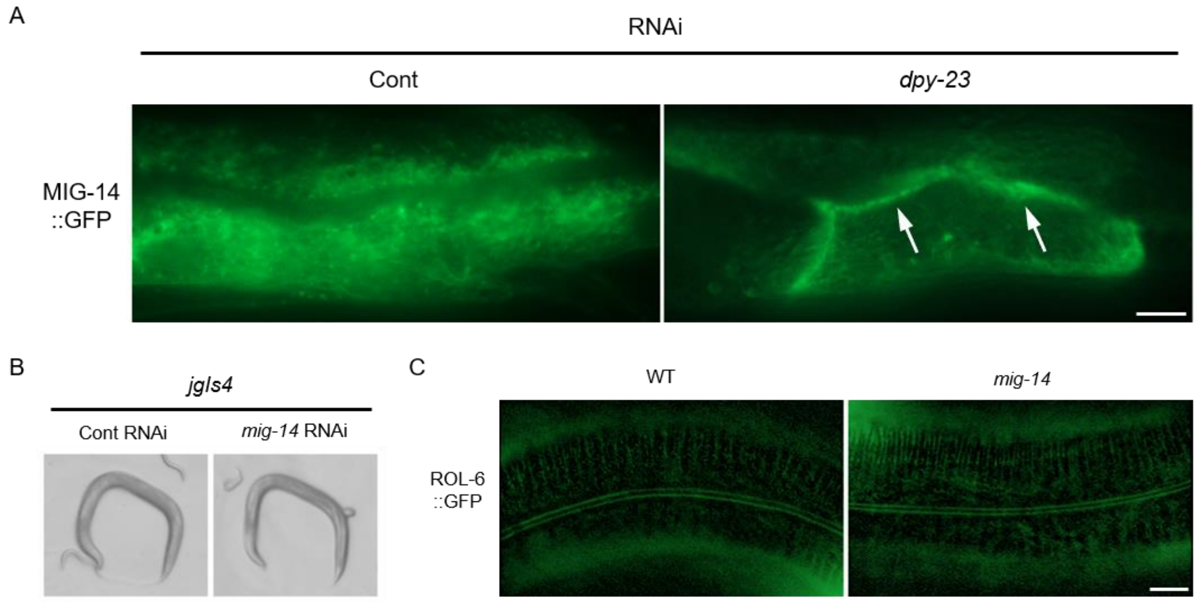

Figure S6. Wnt pathway and DPY-23 in the cuticle. (A) The transgenic strain expressing MIG-14::GFP was observed after *dpy-23* knockdown by RNAi to examine the regulation of MIG-14/Wntless recycling by DPY-23. (B) The Rol strain (*jgIs4*) was fed with *mig-14* RNAi to examine the involvement of the Wnt pathway in the cuticle formation. Arrows indicate the accumulated MIG-14::GFP at the plasma membrane of the intestinal cell. (C) ROL-6::GFP expression was observed in the wild type and the *mig-14* mutant to examine the cuticle structure. Scale bars= 10 μm.

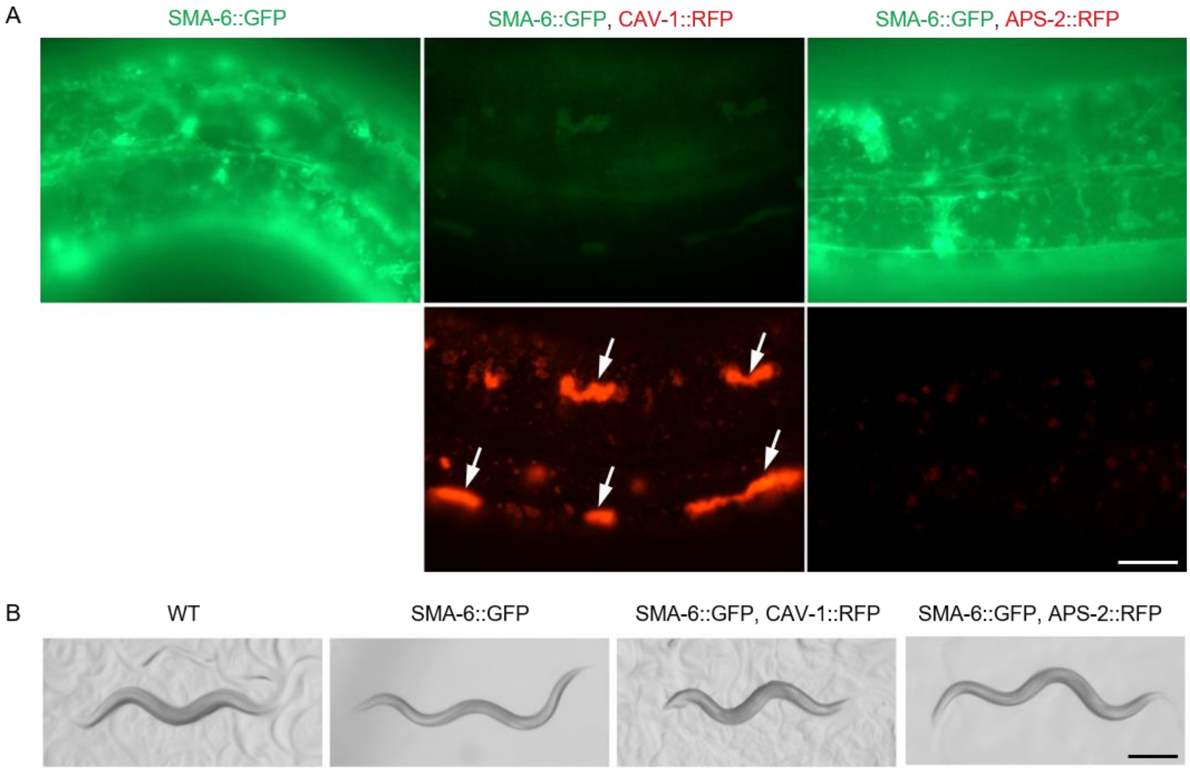

Figure S7. SMA-6 expression by CAV-1 or APS-2 overexpression. (A) The transgenic strains expressing SMA-6::GFP, SMA-6::GFP with CAV-1::tagRFP, and SMA-6::GFP with APS-2::tagRFP by the Y37A1B.5 promoter were observed. Arrows indicate aggregated CAV-1::tagRFP. RFP means tagRFP. Scale bars= 10 μm. (B) The wild-type and transgenic strains expressing indicated proteins in the hypodermis by the Y37A1B.5 promoter were observed using a dissecting microscope. Scale bars= 200 μm.
